# Commentary: Joint and independent associations of dietary vitamin intake and prevalence of cardiovascular disease in chronic kidney disease subjects: a cross-sectional analysis

**DOI:** 10.3389/fnut.2025.1631331

**Published:** 2025-07-14

**Authors:** Wu Zezhi, Wang Huishu, Li Xiangming, Xie Shaoting

**Affiliations:** Guangzhou University of Chinese Medicine Affiliated Shantou Hospital of Traditional Chinese Medicine, Shantou, Guangdong, China

**Keywords:** National Health and Nutrition Examination Survey (NHANES), chronic kidney disease (CKD), cardiovascular disease (CVD), cross-sectional analysis, commentary

We, read with great interest the article titled “Joint and independent associations of dietary vitamin intake and prevalence of cardiovascular disease in chronic kidney disease subjects: a cross-sectional analysis” by Wang et al. (1). By leveraging NHANES data, the study provides valuable insights into the protective roles of vitamins B6, E, and multivitamin co-exposure against cardiovascular disease (CVD) in chronic kidney disease (CKD) populations. The authors should be commended for addressing a critical intersection of nutrition, renal disease, and cardiovascular health. While the authors have thoughtfully acknowledged limitations, we offer additional perspectives to contextualize the findings and guide future research.

First, although NHANES provides nationally representative U.S. data, extrapolation to non-U.S. populations may be limited by differences in dietary patterns, genetic predispositions, and healthcare practices. For instance, vitamin D synthesis varies by latitude and skin pigmentation (2), which could influence CKD outcomes. Furthermore, while the study adjusted for key covariates, unmeasured factors such as systemic inflammation (3), or gut microbiota composition (4) might confound the observed associations. Emerging evidence suggests that gut dysbiosis in CKD patients impairs vitamin K metabolism and exacerbates vascular calcification (5)—a pathway not explored here. Future studies should include biomarkers such as homocysteine or markers of oxidative stress, or dietary pattern analysis using the Mediterranean diet score to elucidate these interactions.

Second, the reliance on 24-h dietary recall introduces potential recall bias and may not reflect long-term intake. While the use of Bayesian kernel machine regression (BKMR) to model multivitamin interactions is innovative, The use of supplements such as over-the-counter multivitamins or CKD-specific formulations is excluded, which limits exposure assessment. Additionally, genetic polymorphisms affecting vitamin metabolism (6) might modulate the observed associations. Integrating genomic data or polygenic risk scores could identify subgroups benefiting most from vitamin interventions.

Third, CKD patients often use medications that alter vitamin absorption or metabolism. For example, statins may synergize with vitamin E to reduce oxidative stress (7), while phosphate binders could impair fat-soluble vitamin uptake (8). The study did not address these interactions, nor did it explore comorbidities like diabetes-related oxidative stress, which may exacerbate CVD risk independently of vitamin intake (9, 10). Future analyses incorporating medication histories and comorbidity stratification could refine clinical recommendations.

Fourth, the cross-sectional design precludes causal conclusions, as reverse causality remains plausible. Longitudinal studies tracking vitamin intake and CVD incidence in CKD cohorts would strengthen temporal inferences. Mendelian randomization could also mitigate confounding by leveraging genetic variants as instrumental variables for vitamin exposure. Additionally, sensitivity analyses using propensity score matching or machine learning approaches might enhance robustness.

Finally, this study underscores the need for personalized dietary strategies in CKD management. For instance, vitamin B6 and E intake thresholds may require adjustment based on CKD stage or dialysis status. Public health initiatives should prioritize targeted nutritional education for CKD patients, particularly those with limited healthcare access. Further research should evaluate whether current guidelines sufficiently address skeletal and cardiovascular risks in this population.

In conclusion, Wang et al. have made a significant contribution to understanding dietary vitamin roles in CKD-related CVD. Our suggestions aim to support future investigations into mechanistic pathways, genetic interactions, and real-world clinical applicability. Such efforts will ultimately inform integrated care strategies to improve outcomes in this high-risk population.
